# Enlargement of One of the Ethmoidal Cells, Etc.

**Published:** 1852-03

**Authors:** Daniel Brainard

**Affiliations:** Prof. of Surgery in Rush Medical College, etc.; Chicago


### ARTICLE VI.

*Case of enlargement of one the ethmoidal cells resembling exostosis of the Orbit. Operation---Recovery.* By Daniel Brainard, M. D., Prof. of Surgery in Rush Medical College, etc.

May 3d, 1851. Murphy, a robust young man from Bureau Co., Ills., aged about 20 years, applied to me on account of a tumor in the orbit, causing a protrusion of the right eye. The globe of the eye was pressed forwards so that the lids could be closed with difficulty and outward so as to come firmly in contact with the external margin of the orbit. At the internal canthus was felt a hard bony tumor extending from the side of the nose outward three-fourths of an inch. It was about two inches in length and curved to conform to the shape of the orbit.

It was first perceived about three years previously, had steadily increased in size, and been unattended by pain, or any discharge from the nostril. Movements and function of the eye perfect. As it was on the point of destroying the usefulness of the eye and had already produced great deformity I determined to attempt its removal.

Being prepared with suitable instruments for removing an exostosis, I raised the integuments by a semi-lunar incision and was proceeding to denude it when carrying the knife with force against the external side it penetrated a bony shell and revealed its contents which was of thick yellow mucous like the inspissated mucous of the nostril. The bone scissors and gouge were then employed to remove the anterior wall when a cavity of the size of a hens egg was revealed. It extended behind the root of the nose to the inner side of the left orbit backwards about two inches, was filled with tenacious yellow mucous without odor and lined with fine polished mucous membrane perfectly healthy. Next the globe of the eye the wall was very thin.

A free opening was made into the nostril at the lower part of the cavity and a tent inserted to be withdrawn when desired, by the nostril, and the wound closed with stitches.

On examining the contents and finding it inspissated mucous the nature of the case became obvious; it was an enlarged ethmoidal cell distended to its present size by the secretion of mucous prevented from escaping by the closure of its orifice. How long it had existed before being detected it is impossible to say, it is not unlikely it may have been congenital. It is obvious that an early opening from the nostril would have arrested its growth but its true nature had not been suspected any one who had examined it and I am not aware that a similar case has been noticed before.

The external wound healed in a week and the patient returned home perfectly cured with the exception of a derangement of the lachrymal apparatus which allowed the tears to flow over the cheek.

*Chicago, Jan.* 31, 1852.

